# Design and study of ultrasound‐based automatic patient movement monitoring device for quantifying the intrafraction motion during teletherapy treatment

**DOI:** 10.1120/jacmp.v13i6.3709

**Published:** 2012-11-08

**Authors:** S. Senthilkumar, R. Vinothraj

**Affiliations:** ^1^ Dept. of Radiotherapy Madurai Medical College & Govt. Rajaji Hospital Madurai India; ^2^ Dept. of Physics Bharathiar University Coimbatore Tamil Nadu India

**Keywords:** ultrasound, radiotherapy, UPMMD, detector, cancer

## Abstract

The aim of the present study is to fabricate indigenously ultrasonic‐based automatic patient's movement monitoring device (UPMMD) that immediately halts teletherapy treatment if a patient moves, claiming accurate field treatment. The device consists of circuit board, magnetic attachment device, LED indicator, speaker, and ultrasonic emitter and receiver, which are placed on either side of the treatment table. The ultrasonic emitter produces the ultrasound waves and the receiver accepts the signal from the patient. When the patient moves, the receiver activates the circuit, an audible warning sound will be produced in the treatment console room alerting the technologist to stop treatment. Simultaneously, the electrical circuit to the teletherapy machine will be interrupted and radiation will be halted. The device and alarm system can detect patient movements with a sensitivity of about 1 mm. Our results indicate that, in spite of its low‐cost, low‐power, high‐precision, nonintrusive, light weight, reusable and simplicity features, UPMMD is highly sensitive and offers accurate measurements. Furthermore, UPMMD is patient‐friendly and requires minimal user training. This study revealed that the device can prevent the patient's normal tissues from unnecessary radiation exposure, and also it is helpful to deliver the radiation to the correct tumor location. Using this alarming system the patient can be repositioned after interrupting the treatment machine manually. It also enables the technologists to do their work more efficiently.

PACS number: 87.53.Dq

## I. INTRODUCTION

The success of radiation therapy is not only to control local tumor growth by delivering a desired dose to the tumor, but also to minimize damage to surrounding healthy tissues. Especially, if there are critical structures around the tumor, both doses delivered to the target and to nearby nontarget organs are the important issues in radiation therapy.^1^, ^3^ In order to achieve higher tumor dose without increasing deleterious complications, tighter treatment margins are required. The proximity of the target to critical organs makes dose escalation challenging, with successful escalation possible by conforming the radiation field to a well‐specified target volume.^(4)^ Inaccurate patient positioning, patient's movements, and organ motion during treatment are the three main reasons for variation of treatment volume during the course of radiation therapy. Patient positioning, or setup error, is the difference in patient positioning between planning and treatment. Patient's organ movements deal with the day‐to‐day changes in the clinical target volume (CTV) position.^5^, ^8^

Patient position monitoring is highly essential to ensure the accurate delivery of radiation therapy treatments. The degree of accuracy for the delivery of tumor dose is recommended to be within ±5% during the treatment, according to the ICRU in Report 24.^9^, ^10^ It is very difficult to achieve, such high‐dose accuracy due to patient immobilization, target localization, and organ motion. Vigilant action on patient movement serves two primary purposes: (1) it ensures that a patient who inadvertently moves during treatment will not receive improper dose; and (2) it ensures that radiation is properly delivered in spite of involuntary patient movements (e.g., breathing). To solve these problems, several approaches have been developed for improving target localization in external beam radiation therapy (EBRT).^11^, ^19^

Patient positioning and immobilization are said to be the most crucial aspects of accurate treatment delivery in EBRT. Immobilization devices allow accurate targeting in radiation therapy treatment. Immobilization devices such as moulds, casts, headrests, and other devices, are used to reduce setup error and patient movement during treatment.^(20)^

The aim of the present study is to fabricate indigenously low‐cost ultrasonic‐based automatic patient's movement monitoring device (UPMMD). The ultrasound frequency range chosen for this device is around 42 kHz based on its high reflection co‐efficient. We have fabricated a sensor device that immediately halts teletherapy treatment if a patient moves, beyond a preset value, claiming accurate field placement and dose delivery. This device is an electronic compact device, which is small in size, low‐power, high‐precision, nonintrusive, low‐cost, lightweight and reusable. UPMMD can be directly attached to the patient to report change in position without any imaging generated by an auxiliary setup using ionizing radiation. This device is very helpful in monitoring the movement of patients during EBRT with or without immobilization device. The number of sensors can easily be expanded in order to monitor movement of multiple body locations simultaneously.

## II. MATERIALS AND METHODS

### A. Construction of UPMMD

**Figure 1:**
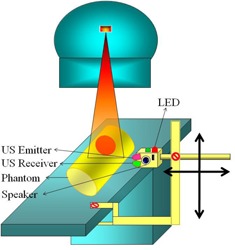
Experimental setup of the UPMMD sensor device.

The UPMMD consists of two main parts: an ultrasound transmitter and receiver. The UPMMD is attached to the treatment couch of the radiotherapy machine with the help of a permanent magnet and screws. It can be easily attached and detached from the couch. The height of the UPMMD can be varied according to the patient's body girth. Figure 1 shows the experimental setup of the UPMMD in radiotherapy machine.

### B. Working principle of UPMMD

**Figure 2:**
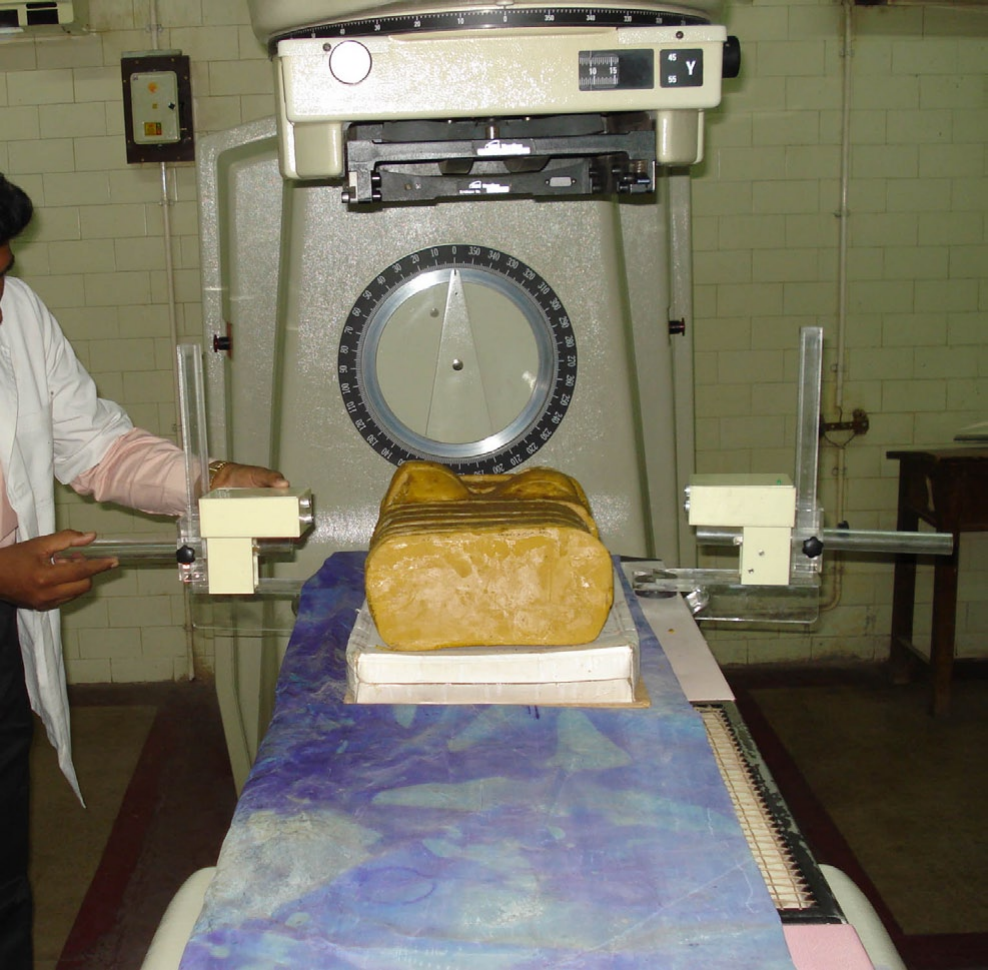
Photograph of the UPMMD sensor device experimental setup in radiotherapy machine.

**Figure 3:**
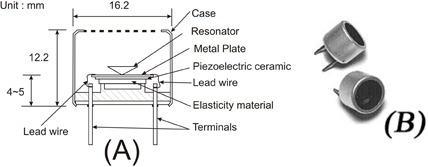
The ultrasonic transducer block diagram (a) and diagrammatic representation of the transducer (b).

**Figure 4:**
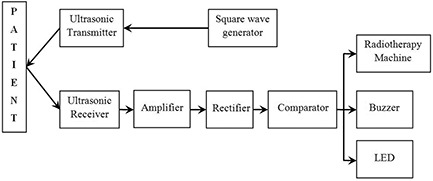
The block diagram of the UPMMD sensor device.

Figure 2 shows the photograph of the UPMMD sensor device experimental setup in radiotherapy machine with phantom. The UPMMD functions on the principle of reflection of ultrasonic waves. Ultrasonic waves are defined as longitudinal pressure waves in the medium in which they are traveling. Objects, whose dimensions are larger than the wavelength of the impinging sound, reflect them; the reflected waves are called echo and these waves are inaudible to human ears, but are highly energetic. These waves travel with the speed of $330\;{\text{ms}}^{‐1}$, equivalent to the speed of sound in air medium, and the time elapse between the emission of ultrasonic waves and the reception of the echo is recorded. The ultrasonic waves travel twice the distance between the source and the patient. The actual distance between the source and patient is d/2 meters, where d is the distance travelled by the ultrasonic signal. The specification of the ultrasonic sensor are found in Table 1. (Figure 3a) shows the parts of the ultrasonic transducer and diagrammatic representation of the transducer is shown in (Fig. 3b). Figure 4 shows the block diagram of the UPMMD sensor device.

**Table 1:** The specification of the ultrasonic sensor.

*Item*	*Specification*
Resonant frequency (KHz)	40
Sound pressure level (dB)	115<
Sensitivity (dB)	‐64<
Size (mm) Diameter	16.2
Height	12.2
Terminal Interval	10.0

### C. Ultrasonic transmitter

The ultrasonic transmitter circuit uses an IC 555 timer (Signetics Corporation, Paju‐si Gyungki‐do, Republic of Korea) to generate square waves and it can be used as a free running multivibrator. The IC 555 timer has two comparators, an RS flip‐flop, and a discharge transistor. By using this IC, the square waves of 40–42 kHz frequency can be generated and fed to the ultrasonic transmitter. The values of components used to generate the square waves at the frequency range between 40 and 42 kHz are $R_{A}=14.7\;k\Omega$, $R_{B}=18\;k\Omega$ and C=680 nF. Hence, the frequency of oscillation $f=1.45/\;((R_{A}2R_{B})*C)=42\;\text{kHz}$.

### D. Ultrasonic receiver

The ultrasonic receiver circuit is used to sense the ultrasonic signals. This circuit consists of a two‐stage amplifier, a rectifier, and a comparator in its inverting mode. Output of the comparator is connected to a buzzer alarm, a red light emitting diode (LED) indicator, as well as to the treatment machine control panel. The ultrasonic receiver accepts the reflected ultrasonic signal from the patient and converts it into electrical variations. Patient is positioned for the treatment on the couch. Now the sensor device is attached to the treatment table which is near to the treatment area. It is initially indicated by the glow of green LED. It identifies the signal reflected from the patient. This signal strength, which was a function of a distance, could be considered as an initial position of the patient. Signal strength is calibrated as a function of a distance by means of potentiometer. The sensor measures the distance from its initial position with respect to the movement of the patient, away or towards the sensor. If the patient moved from the threshold limit, the output signal of UPMMD either simultaneously is sent to the buzzer, LED, and radiotherapy machine, or individual alerts can also be possible, per the arrangement of the UPMMD setup. Above settings took less than a minute for each sensor in UPMMD. The buzzer is connected to the circuit which is placed in the control console of the radiotherapy room. The output of the UPMMD is connected serially to the radiotherapy machine, similar to a door interlocking mechanism. During the radiotherapy treatment, if the door opens, the machine will stop the emission of radiation. Likewise, when the UPMMD threshold exceeds the preset value, UPMMD makes the radiotherapy machine stop the emission of radiation. The transmitter and receiver circuit is powered by a 9 volt battery or an eliminator.

The square output produced by IC 555 timer is connected to the input terminal of the ultrasonic transmitter. The output of the receiver is amplified by feeding it into the base of the first transistor. It is again amplified by a second transistor to make the signal as in original phase. The bypass capacitor is used as a filter to reduce the noise problems. Then the amplified signal is compared using a comparator and the range can be adjusted with the help of a potentiometer. This potentiometer helps to vary the sensitivity of the device so that it can be used for different sites of cancer. It helps to meet out the different threshold levels depending on the location of the tumor. In the same way, the other ultrasonic sensor is constructed. The logical output of the two sensors is given to two inputs of an OR gate (IC 7432). When any one of the inputs is high, the OR gate will give logical high output, which can be used to trigger the indicator and the buzzer connected at the output terminal.

The UPMMD can be interfaced to the Cobalt ‐60, linear accelerator machine and an audible alarm can notify technicians of any undesired patient movement. The accuracy of the UPMMD is 1 mm of patient movement. It is possible to notify the patient of any excessive movement. Furthermore, UPMMD can guide the patient to shift to the original reference position through a series of audible commands. The radiotherapy machine will be halted automatically and after repositioning the patient, the treatment can be carried out for the remaining dose.

### E. Phantom study

The phantom study has been performed using the in‐house bees wax thoracic phantom to analyze the accuracy and sensitivity of the UPMMD at various positions. The UPMMD is mounted on both sides of the couch using screws and magnetic support to increase the rigidity of the device. The whole device can be fixed at anywhere on both the sides of the couch. The sensor head is adjustable in transverse and up and down directions, so that, the movement of the device is enabled in all directions. The phantom is placed over the couch in the exact treatment position and then the UPMMD is attached near the treatment area. A specific ultrasound target area is selected in such a way that the ultrasonic waves get reflected from the phantom. When the signal of selected intensity is received from the patient's body, the green LED glows. The threshold level of the UPMMD can be adjusted by varying the potentiometer value even when the movement of the patient treatment area varies from 1 mm onwards.

The phantom study was done to find the accuracy and sensitivity of the UPMMD. Measuring scales in millimeters (mm) were fixed on the table on bottom and top sides of the phantom. Center of phantom had been marked as the reference point on the scale on both sides of the phantom. The distance between the UPMMD and the phantom was standardized by adjusting the potentiometer. To decide the threshold level of the UPMMD, the phantom is manually moved 1 mm away from the UPMMD. Since the phantom is moved from the initial position, the alarm is raised and the red LED glows. In order to calibrate the 1 mm threshold of the device, the potentiometer was adjusted until the alarm stops and also the indication changed from red LED to green LED. This particular position of the potentiometer was marked. This is the reference point for the 1 mm threshold level. Similar procedure was repeated to calibrate the threshold levels up to 10 mm at an interval of 1 mm.

**Figure 5:**
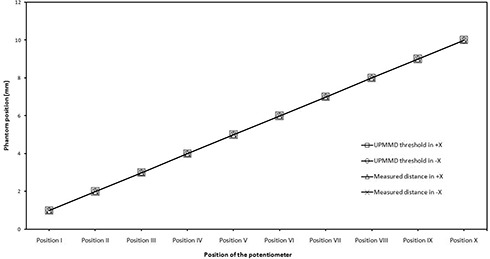
Position of the potentiometer and phantom movement for a fixed threshold levels in +X and ‐X directions for different phantom positions from UPMMD, and the measured value of the phantom position when the sensor gets triggered.

To check the sensitivity of the UPMMD, the phantom was placed in the treatment position and the UPMMD was fixed in the sensing position. The threshold was fixed for 1 mm. The alarm was raised when the phantom moved 1 mm away from the initial position along the +X direction. The same procedure was carried out for ‐X direction. Different positions of the potentiometer and the different phantom positions for a fixed threshold levels in +X and ‐X directions are shown in Fig. 5.

### E. Patient study

The patient was positioned in the treatment table according to the treatment position. The UPMMD was attached near to the treatment area. The UPMMD is activated in the initial position. The initial position of the patient is observed by light field and marked by tattoos. If the patient moves, the light field varies and also the tattoos. The error is measured by the distance between field light and patient marking. For the patient study, the UPMMD device was utilized for observing the movement of 210 different types of cancer patients during the radiotherapy treatment.

## III. RESULTS & DISCUSSION

The ultrasonic emitter produces the ultrasonic waves and the receiver accepts the reflected ultrasonic signal from the patient. When a patient moves, the receiver activates the circuit; an audible warning alarm is produced in the console room. Through real‐time measurements, an audible alarm can alert the radiation technologist to stop the treatment if the user‐defined positional threshold is violated. Simultaneously, the electrical circuit to the teletherapy machine will be interrupted and radiation will be halted.

The UPMMD equipment was utilized for observing the movement of 210 patients (65 were cervix cancer, 20 breast cancer, 25 head‐and‐neck cancer, 12 lung cancer, 18 esophagus cancer, 22 brain tumor, 12 pediatric patients, 6 unconscious patients, 3 soft tissue sarcoma (STS) of leg, 2 STS of arm, 2 Hodgkin's disease, 6 spine tumor, 6 rectum cancer, 8 bladder cancer, and 3 maxilla cancer patients). In this study, immobilization devices have not been used for the patients during the treatment.

During the radiotherapy treatment, the patient's movements were detected by the UPMMD; as a result, the machine turns to the standby mode, as well as alarming device in the control console. Thereby, the emission of radiation from the machine gets stopped. When we entered into the treatment room and checked the patient's position, the patient had moved a distance ranging from 6 mm to 15 mm laterally from the actual treatment area. Hence, the analysis has been made to observe the patient's movements for the different types of cancer. The observed data are presented in Table 2. Treatment was given to 65 Ca. Cervix‐affected patients, among them 8 patients were moved from the original position due to the patient adjusting for comfortable position. The movements for head and neck cancer affected patients were identified due to the neck pain, swallow, cough, and breathing.

**Table 2:** Patient movements observed for different types of cancer.

*Type of Cancer*	*Number of Patients*				
	*Total*	*No. With Movement*	*No. Without Movement*	*Distance Moved from the Initial Position (mm)*	*Reason for Patient Movements*
Ca. Cervix	65	8	57	6	Adjusting for comfortable position
Ca. Breast	20	5	15	10	Pain of hand
Head and Neck	25	20	5	12	Neck pain / Swallow
Ca. Lung	12	7	5	9	Cough/Sneezing
Ca. Esophagus	18	8	10	6	Cough
Brain Tumor	22	11	11	10	Pain
Pediatric	12	9	3	15	Child behavior
Unconscious	6	6	0	15	Unconsciousness
STS (Leg)	3	2	1	10	Pain of leg
STS (Arm)	2	2	0	10	Pain of arm
Hodgkin's Disease	2	2	0	12	Cough
Spine Tumor	6	1	5	10	Pain / Breathing
Ca. Rectum	6	2	4	11	Pain /Prone position ‐ Uncomfortable
Ca. Bladder	8	4	4	7	Abdominal movement / pain
Ca. Maxilla	3	2	1	15	Neck pain /Face movement

Among the 25 breast cancer affected patients, 5 moved their hands due to the pain. Brain cancer patients have moved due to the unconscious and pain of the body. The patients affected by Ca. Lung and Ca. Esophagus have changed their positions due to unexpected sneezing and cough. Fifty percent of brain tumor patients moved from their initial positions due to the unconsciousness and pain of the body. Due to the child behavior of pediatric patient, movements were observed for 9 patients among 12. Movements were observed for all the patients who were in unconscious state and affected by Hodgkin's disease due to cough and sneezing. STS (leg) and STS (arm) patients moved due to the pain of the leg and arm, respectively. Due to the pain and breathing, spine tumor patient moved. Some of the Ca. Rectum and Ca. Bladder cancer patients were not able to stay in their original positions due to such reasons as prone position, abdominal movement, pain of the body, and uncomfortableness. Cancer Maxilla patients moved due to the neck pain and facial movement. UPMMD threshold can be fixed by the operator depending upon the staging and site of the tumor. For example, minimum threshold is fixed for more sensitive organs (eye) and increased threshold is fixed for palliative cases. The device and alarm system can detect the patient movement with a sensitivity of about 1 mm.

It can be concluded that among the 210 patients, 89 (8 cervix, 5 breast, 20 head and neck, 7 lung, 8 esophagus, 11 brain tumor, 9 pediatric, 6 unconscious, 2 STS (leg), 2 STS (arm), 2 Hodgkin's disease, 1 spine tumor, 2 rectum, 4 bladder, and 2 maxilla) cancer patients moved from their set positions during the treatment by more than 3 mm, whereas the rest received the radiation without movements. During the treatment, the interruption of radiation has occurred due to the patient's movement from 1 to 15 mm laterally from the actual treatment area. It is possible to restart the machine only after repositioning the patient. So, the patient was repositioned exactly to the previous treatment position and then the machine was switched ON for radiation treatment. A large number of interruptions were found in the head and neck, pediatric, and the unconscious patients. Therefore, it would be more effective to sense the patient's movement during the radiotherapy treatment in two ways: one is to turn the radiotherapy machine in standby mode, and the second is to alert the radiation technologist by audio speaker and by a visual indicator.

The use of patient movement monitoring techniques discussed in these studies show great promise to the improvement and progression of radiation therapy techniques. The need for accurate patient positioning through the entire treatment for cancer patient with the use of monitoring device is evident of monitoring the patient's movement effectively. Individuals must be informed about the importance of immobilization devices in order to understand that more focus must be placed in continuing to improve the technology behind them. Improved immobilization techniques and devices can contribute to smaller treatment volumes, higher controlled doses, lower patient side effects and, ultimately, a higher cure rate.

The UPMMD device is constructed in order to fulfill the need for accurate patient position monitoring during the entire treatment. This device successfully monitors the patient movement and alerts the radiotherapy technologist. This device can be used as an additional tool for the patients undergoing radiotherapy treatment with or without immobilization devices.

The present work is based on lateral movement of the patient during the radiotherapy treatment. Further work will be carried out in future to focus on the N‐dimensional movement of the patient. UPMMD is capable of sensing the movement of the patient in all directions by using multiple sensors in the all other desirable directions.

## IV. CONCLUSIONS

An inexpensive automatic patient movement monitoring sensor device was successfully fabricated using ultrasound, and patient's movements were quantitatively analyzed, using the UPMMD device. The UPMMD is an electronic compact device which is small in size, high‐precision and light in weight. This real‐time ultrasound‐based patient movement monitoring device is easy to handle in verifying positional change in patients undergoing radiation treatment. The sensitivity of this device can be varied for different sites of cancers in the human body. Although patients are monitored closely by radiation technologists using closed‐circuit television at the hospital, slight movements may not be seen, especially during the time when a technologist walks from the treatment table to the EBRT console located in an adjacent room. Hence, this device can prevent the patient's normal tissues from unnecessary radiation exposure and also it is helpful to deliver the radiation to the correct tumor location. Another method to control the motion is using an audible device. The raising sound alerts the radiation technologists. By using this alarm system, the patient can be repositioned after interrupting the treatment machine manually. It also enables the technologists to do their work more efficiently, as they don't have to continuously monitor patients with as much scrutiny. In addition to detecting small, inadvertent patient movements, UPMMD can be used in conjunction with different imaging modalities also.

Studies were conducted on phantom and 210 patients with different types of cancers. Our preliminary clinical test results indicate that UPMMD is highly reliable and can accurately report smaller movements of the patients. The results demonstrated that the device was able to detect patient's movements with the sensitivity of about 1 mm. An additional benefit is that it has reduced the tension and stress associated with treating patients who are not immobilized. Furthermore, we showed that under high energy radiation UPMMD performs well with no degradation to the ultrasound signal. Although the potential capabilities of UPMMD suggest an improvement over current practice, questions of logistics, durability, and clinical functionality still remain. The design of UPMMD is highly flexible. It can be connected to multiple detectors, allowing detection of the position change in different parts of the patient's body. Our results indicate that, in spite of its low‐cost and simplicity, UPMMD is highly sensitive and offers better treatment with less deviation from the control. Furthermore, UPMMD is user‐friendly and requires only minimal training. The versatile architecture of UPMMD makes it potentially suitable for variety of applications with no major changes in machine setup or hardware setup.

This study was limited to description of the development of UPMMD and an initial evaluation of its feasibility for clinical use. More detailed clinical studies are required to quantify the system's practicality and effectiveness in a simulated environment. Important advantageous features of UPMMD include ease of use on patients, minimal user training, and needs no change in the typical patient and the machine setup. So, the UPMMD can potentially be used to monitor and control the patient's movement during EBRT.

## References

[1] Dobbs J . Practical radiotherapy planning, 2nd edition. London, Melbourne: Hodder & Arnold; 1992.

[2] Senthilkumar S and Ramakrishnan V . In‐house auto cutoff sensor device for radiotherapy machine to monitor patient movements. J Appl Clin Med Phys. 2008;9(3):82–89. 10.1120/jacmp.v9i3.2800 PMC5722305 18716594

[3] Khan FM and Potish RA . Treatment planning in radiation oncology. Baltimore, MD: Williams & Wilkins; 1998.

[4] Ding M , Li J , Deng J , Fourkal E , Ma CM . Dose correlation for thoracic motion in radiation therapy of breast cancer. Med Phys. 2003;30(9):2520–29. 14528974 10.1118/1.1603744

[5] Onishi H , Kuriyama K , Komiyama T , et al. CT evaluation of patient deep inspiration self‐breath‐holding: how precisely can patients reproduce the tumor position in the absence of respiratory monitoring devices? Med Phys. 2003;30(6):1183–87. 12852542 10.1118/1.1570372

[6] Langen KM and Jones DT . Organ motion and its management. Int J Radiat Oncol Biol Phys. 2001;50(1):265–78. 11316572 10.1016/s0360-3016(01)01453-5

[7] International Commission on Radiation Units and Measurements . Prescribing, recording and reporting photon beam therapy. ICRU Report 50. Bethesda, MD: ICRU; 1993.

[8] International Commission on Radiation Units and Measurements . Prescribing, recording and reporting photon beam therapy (supplement to ICRU Report 50). ICRU Report 62. Bethesda, MD: ICRU; 1999.

[9] George R , Keall PJ , Kini VR , et al. Quantifying the effect of intrafraction motion during breast IMRT planning and dose delivery. Med Phys. 2003;30(4):552–62. 12722807 10.1118/1.1543151

[10] International Commission on Radiation Units and Measurements . Determination of absorbed dose in a patient irradiated by beams of X or gamma rays in radiotherapy procedures. ICRU Report No.24. Washington, DC: ICRU; 1976.

[11] Mostaar A , Allahverdi M , Shahriari M . Application of MCNP4C Monte Carlo code in radiation dosimetry in heterogeneous phantom. Iran J Radiat Res. 2003;1(3):143–49.

[12] Ryken TC , Meeks SL , Pennington EC , et al. Initial clinical experience with frameless stereotactic radiosurgery: analysis of accuracy and feasibility. Int J Radiat Oncol Biol Phys. 2001;51(4):1152–58. 11704340 10.1016/s0360-3016(01)01756-4

[13] Lattanzi J , McNeeley S , Hanlon A , Schultheiss TE , Hanks GE . Ultrasound‐based stereotactic guidance of precision conformal external beam radiation therapy in clinically localized prostate cancer. Urology. 2000;55(1):73–78. 10654898 10.1016/s0090-4295(99)00389-1

[14] Bouchet LG , Meeks SL , Bova FJ , Buatti JM , Friedman WA . Three‐dimensional ultrasound image guidance for high‐precision extracranial radiotherapy. Radiosurgery. 2002;4:262–78.

[15] Phillips MH , Singer K , Miller E , Stelzer K . Commissioning an image‐guided localization system for radiotherapy. Int J Radiat Oncol Biol Phys. 2000;48(1):267–76. 10924998 10.1016/s0360-3016(00)00581-2

[16] Tome WA , Meeks SL , Orton NP , Bouchet LG , Bova FJ . Commissioning and quality assurance of an optically guided three‐dimensional ultrasound target localization system for radiotherapy. Med Phys. 2002;29(8):1781–88. 12201425 10.1118/1.1494835

[17] Shimizu S , Shirato H , Kitamura K , et al. Use of an implanted marker and real‐time tracking of the marker for the positioning of prostate and bladder cancers. Int J Radiat Oncol Biol Phys. 2000;48(5):1591–97. 11121666 10.1016/s0360-3016(00)00809-9

[18] Litzenberg D , Dawson AA , Sandler H , et al. Daily prostate targeting using implanted radiopaque markers. Int J Radiat Oncol Biol Phys. 2002;52(3):699–703. 11849792 10.1016/s0360-3016(01)02654-2

[19] Murphy MJ . Fiducial‐based targeting accuracy for external‐beam radiotherapy. Med Phys. 2002;29(3):334–44. 11929016 10.1118/1.1448823

[20] Yan H , Yin FF , Kim JH . A phantom study on the positioning accuracy of the Novalis Body system. Med Phys. 2003;30(12):3052–60. 14713071 10.1118/1.1626122

